# Interdepartmental approach to early malocclusion management: A pediatric and orthodontic collaborative study

**DOI:** 10.6026/973206300222890

**Published:** 2026-05-31

**Authors:** Jugal Burela, Kanchan Sharma, Nikita Pal, Yeptasam Yasmin, Bhupendra Singh, Sabyasachi Chakraborty

**Affiliations:** 1 Department of Orthodontics and Dentofacial Orthopaedics, Awadh Dental College and Hospital, Jamshedpur, Jharkhand, India; 2 Department of Orthodontics and Dentofacial Orthopaedics, ITS Centre for Dental Studies and Research, Muradnagar, Ghaziabad, Uttar Pradesh, India; 3 Department of Conservative Dentistry and Endodontics, Haldia Institute of Dental Sciences and Research, Haldia, West Bengal, India

**Keywords:** Early malocclusion, pediatric dentistry, orthodontics, interdepartmental approach, preventive orthodontics, interceptive treatment, occlusal development, child dental care, collaborative management, Index of Orthodontic Treatment Need (IOTN), behavioral compliance

## Abstract

Malocclusion can result in temporomandibular joint dysfunction, impaired speech, impaired mastication, aberrant craniofacial development and psychosocial difficulties. Therefore, it is of interest to assess early malocclusion in 50 children ages 6 to 12. Children were monitored every 6-8 weeks and received preventive, interceptive, or observational care based on joint assessments. The findings revealed a 78% improvement in occlusion, along with high parental satisfaction (92%) and compliance (88%). A statistically significant improvement was noted (p < 0.05). Thus, cooperative early intervention can improve occlusal outcomes and lower the need for orthodontic treatment in the future.

## Background:

One of the most common dental conditions affecting developing children globally is malocclusion, which has consequences that go far beyond appearance. Untreated malocclusion can result in temporomandibular joint dysfunction, impaired speech, impaired mastication, aberrant craniofacial development and psychosocial difficulties that can impact a child's social and self-esteem [1, 2]. Malocclusion has been identified as the third most prevalent oral health issue worldwide, after periodontal disease and dental caries, highlighting its substantial public health burden [3]. Some malocclusions are minor or self-limiting, but others can get worse over time, changing from dental to skeletal in nature and requiring more involved, time-consuming and expensive treatments later in adolescence or adulthood. Early detection and intervention are therefore essential, especially during the mixed dentition period (usually between the ages of 6 and 12), when growth-modifying treatments and interceptive measures are most successful [4, 5]. In addition to improving occlusal relationships, oral function and facial aesthetics, prompt correction during this formative stage significantly lowers the risk of additional complications, minimises the psychosocial impact and lessens the emotional and financial strain of future orthodontic care [6, 7]. Additionally, children are typically more flexible at this age, which improves their cooperation with behavior-modification techniques and orthodontic appliances. Paediatric dentists are in a unique position to identify subtle signs of malocclusion because of their frequent interactions with children starting at a young age. These include open bites, deep bites, crossbites, dental crowding and bad oral habits like mouth breathing, tongue thrusting and thumb sucking and prolonged dummy use [8, 9]. Paediatric dentists are essential in detecting and preventing malocclusion in its early stages through regular screenings and evaluations. Clinical knowledge from both orthodontics and paediatric dentistry can be integrated through a cooperative approach. While orthodontists offer specialised knowledge in biomechanics, skeletal analysis and long-term occlusal planning, paediatric dentists are strong in behaviour management, preventive measures and early habit intervention. These experts can collaborate to create treatment plans that are specific to the child's needs and developmentally appropriate. Early referrals prompt appliance delivery, shared follow-up and ongoing growth and treatment response monitoring are all made possible by interdepartmental collaboration [10, 11]. When working with children who need interceptive treatments-like space maintainers, functional appliances or myofunctional therapy-which all calls for high levels of compliance and constant supervision, this synergy is especially crucial. Paediatric dentists are uniquely qualified to improve patient cooperation, which is crucial for the success of early orthodontic treatment, because they have received training in positive reinforcement, desensitization and anxiety management [12, 13]. Furthermore, a methodical framework for assessing the severity of malocclusion and setting care priorities is offered by the use of standardised diagnostic indices like Angle's classification system and the Index of Orthodontic Treatment Need (IOTN). These tools facilitate the referral process, improve the efficiency of clinical resource allocation and guarantee that children who require the most care receive it promptly [14, 15]. In order to identify children who are at risk and incorporate interceptive orthodontics into public health and school-based programs, research also supports the use of World Health Organisation (WHO) guidelines on occlusal traits in community dental health programs. Particularly in environments with limited resources, these international standards promote consistent screening procedures, early triage and data-driven policy development [16]. Therefore, it is of interest to assess the clinical efficacy and patient-centered results of a structured, team-based care model that combines the fields of orthodontics and paediatric dentistry.

## Methodology:

Over the course of a year, the Departments of Paediatric Dentistry and Orthodontics collaborated to conduct this prospective observational study in a tertiary dental care facility. 50 kids in all, ages 6 to 12, were chosen using particular inclusion and exclusion criteria. Children who showed cooperative behaviour (Frankl rating 3 or 4), had no prior orthodontic treatment and displayed early malocclusion symptoms (such as crowding, anterior or posterior crossbites, or open bite) were included. A history of dental trauma affecting occlusion, systemic conditions influencing dental development, the presence of craniofacial anomalies (such as cleft lip or palate), or current involvement in orthodontic or orthopaedic therapy were among the exclusion criteria. Paediatric dentists conducted the initial screening of patients as part of their regular dental examinations. Children who met the eligibility requirements were sent to the orthodontics department, where paediatric dentists and orthodontists conducted a collaborative clinical assessment. Orthopantomograms (OPG), lateral cephalograms, dental casts, extraoral and intraoral examinations, medical and dental histories and intraoral photos were all part of a standardised examination protocol for every child. The Index of Orthodontic Treatment Need (IOTN), WHO occlusal characteristics and Angle's classification were used to categorise malocclusions. An interdepartmental treatment plan was developed after a thorough evaluation, taking into consideration the child's dental development, growth pattern, behaviouralcompliance and risk of malocclusion progression. Children were divided into three management groups based on this assessment: observation only for minor malocclusions needing periodic monitoring, preventive (like space maintainers or habit-breaking appliances), or interceptive (like crossbite correction or functional appliances). In order to track development, evaluate appliance use and efficacy, assess behavioural compliance and record any issues, each child was monitored at regular intervals of 6-8 weeks. The degree of malocclusion improvement, patient comfort and compliance and parental satisfaction as measured by structured feedback forms were among the outcome measures at the conclusion of the 12-month follow-up. Additionally, the necessity of additional orthodontic treatment was assessed. Microsoft Excel was used to compile the data and SPSS software version 25 was used for analysis. The data was summarised using descriptive statistics like means, frequencies and percentages. Chi-square and paired t-tests were used to compare the results before and after treatment, with a significance level of p < 0.05.

## Results:

A total of 50 children aged between 6 and 12 years were included in the study, with 28 males (56%) and 22 females (44%). The most common age group was 8-10 years, coinciding with the mixed dentition phase. Based on Angle's classification, 60% of participants had Class I malocclusion, 26% had Class II and 14% had Class III. Assessment using the WHO occlusal traits and the Index of Orthodontic Treatment Need (IOTN) showed that 72% (n = 36) had a moderate to high treatment need, while 28% (n = 14) required minimal or borderline treatment (Table 1). Participants were managed through three different approaches: preventive treatment (36%), interceptive treatment (48%) and observation (16%). Over a 12-month follow-up period, 78% of children (n = 39) showed notable improvement in malocclusion, with the interceptive group demonstrating the most significant changes (Table 1). Furthermore, appliance comfort and compliance were high, with 84% of children reporting good tolerance to the appliances and behavioral cooperation, as assessed using the Frankl Rating Scale, improved or remained stable in 88% of cases. Structured feedback indicated that 92% of parents were either satisfied or highly satisfied with the treatment outcomes. However, 10 children (20%) were identified as requiring further, more comprehensive orthodontic care due to persistent skeletal discrepancies (Table 2). Statistical analysis revealed a significant correlation between the type of treatment and occlusal improvement using the Chi-square test (p < 0.05). The paired t-test also confirmed statistically significant improvements in occlusion between baseline and follow-up (p < 0.01); particularly within the interceptive treatment group (Table 2).

## Discussion:

Accurate diagnosis, space analysis and growth assessment are critical to the successful treatment of early malocclusion. Predicting the mesiodistal widths of unerupted canines and premolars is a fundamental component of early orthodontic planning. In order to develop interceptive strategies during the mixed dentition stage and enable space maintenance or regaining procedures when required, Popovich and Thompson's seminal study highlighted the significance of this prediction [16]. These examinations guarantee that erupting permanent teeth line up correctly, free from crowding or impaction. Another essential component of early malocclusion intervention is space management. According to Ngan *et al.* if space maintainers, guided eruption techniques or arch development appliances are detected and treated early on, space issues in the mixed dentition can frequently be fixed without the need for extensive treatment [17]. This is consistent with the current study, in which children with moderate to high treatment needs benefited from the successful implementation of preventive and interceptive measures. Early orthopaedic interventions are ideal for managing maxillary deficiencies, which are frequently associated with Class III malocclusions. Maxillary protraction, with or without rapid palatal expansion, has been shown by Vaughn *et al.* to have a significant impact on promoting favourable skeletal changes in growing children [18]. These methods were found to be helpful in the interceptive cases in the current study and can lessen the need for subsequent orthognathic surgeries when used during the proper growth phase. Important diagnostic data is also provided by the palatal vault's morphology. This supports the structured evaluations carried out in our nterdisciplinary model and highlights the importance of three-dimensional diagnostic aids, particularly in complex or borderline malocclusion cases. Lastly, determining the best time for treatment requires an understanding of craniofacial growth patterns. Facial growth evaluation aids in determining which malocclusion characteristics can be treated early and which call for postponed intervention for skeletal correction, as Vig and Fields thoroughly described [19]. Our strategy, which identified children with noticeable skeletal abnormalities for future orthodontic treatment after growth completion, reflected this idea. Early functional appliance therapy has a well-established track record of success from an orthodontic perspective. When growth potential is high, especially in Class II and crossbite corrections, Zierhut *et al.* address the skeletal and dentoalveolar modifications that can be achieved with such appliances [20]. Similar to this, Bishara *et al.* emphasises the significance of treatment timing, confirming that some interventions lose their effectiveness or necessitate invasive corrections later on if they are postponed [21]. The current study's diagnostic focus on cephalometric and photographic analyses is supported by structural assessments, such as those described by McNamara, which emphasise the importance of skeletal pattern evaluations in early age groups (8-10 years) [22]. Since early orthodontic treatment of malocclusion in children coincides with the peak growth and development of children, the comprehensive management of children's oral and general health cannot be neglected [23]. By taking these observations into account, our research not only supports but also validates the body of research that supports an organised, multidisciplinary and child-centered approach to orthodontic treatment. These early interventions provide a more comprehensive treatment pathway by addressing the emotional, developmental and social aspects of child health in addition to occlusion, supported by multispecialty evaluation and collaboration.

## Conclusion:

We show the efficacy of an interdepartmental approach between orthodontics and paediatric dentistry in the early treatment of childhood malocclusion. Parental satisfaction, high compliance and occlusal outcomes all significantly improved with prompt preventive and interceptive interventions. Collaborative care models are crucial in order to reduce future orthodontic complexity and to promote long-term oral health.

## Figures and Tables

**Table 1:** Treatment outcomes and compliance parameters (n = 50)

**Outcome Parameter**	**Value / Frequency**
Children with malocclusion improvement	39 (78%)
Most effective treatment type	Interceptive (24 children)
Appliance tolerance (positive response)	42 children (84%)
Behavioral compliance improved/stable	44 children (88%)
Minor appliance-related issues	6 children
High parental satisfaction (from feedback forms)	92% of parents
Required further orthodontic intervention	10 children (20%)
Statistical significance (Chi-square)	p < 0.05
Pre-post occlusal score significance (t-test)	p < 0.01

**Table 2:** Clinical and outcome parameters in early malocclusion management study (n = 50)

**Parameter**	**Result**
Total children enrolled	50
Gender distribution	28 males (56%), 22 females (44%)
Most common age group	8-10 years
Angle's classification	Class I: 60%, Class II: 26%, Class III: 14%
IOTN/WHO-based treatment need	Moderate to high: 72%, Minimal: 28%
Treatment type distribution	Preventive: 36%, Interceptive: 48%, Observation: 16%
Improvement in malocclusion	78% (39 children)
Appliance comfort/compliance	84% (42 children)
Behavioral compliance (Frankl rating stable/improved)	88%
Parental satisfaction	92%
Children needing further orthodontic treatment	20% (10 children)
Statistical significance (Chi-square, paired t-test)	p< 0.05, p< 0.01
